# Multicenter clinical performance and comparative evaluation of the LIAISON PLEX Respiratory *Flex* Assay for syndromic detection of viral and bacterial pathogens in nasopharyngeal swabs

**DOI:** 10.1128/jcm.00708-25

**Published:** 2025-11-05

**Authors:** Kaisha Gonzalez, Jennifer Dien Bard, Deanna Becker, Brian Bernier, Suzane Silbert, Jennifer Meece, Janet Farhang

**Affiliations:** 1 Diasorin, Austin, Texas, USA; 2 Department of Pathology and Laboratory Medicine, Children’s Hospital Los Angeles 5116, Los Angeles, California, USA; 3 Keck School of Medicine, University of Southern California 5116, Los Angeles, California, USA; 4 Tampa General Hospital 7829, Tampa, Florida, USA; 5 Marshfield Clinic 513992 , Marshfield, Wisconsin, USA; National Institute of Allergy and Infectious Diseases Division of Intramural Research, Bethesda, Maryland, USA

**Keywords:** PCR, respiratory infections, syndromic testing

## Abstract

The LIAISON PLEX Respiratory *Flex* Assay is a customizable syndromic molecular assay for the qualitative detection of nucleic acids from 14 viral and 5 bacterial respiratory pathogens in nasopharyngeal swabs (NPS). This study evaluated its clinical performance in comparison to established FDA-cleared and standard-of-care molecular diagnostic platforms. A total of 2,099 NPS specimens were prospectively and retrospectively collected. Positive Percent Agreement (PPA), Negative Percent Agreement (NPA), and diagnostic accuracy were calculated across 19 targets. The assay was benchmarked against SOC assays, including Cepheid Xpert Xpress CoV-2/Flu/RSV Plus, Abbott Alinity m Resp-4-Plex, and BioFire RP 2.1 panel. Agreement metrics, kappa coefficients, and discordance analysis were used to assess the comparative performance. For bacterial targets, PPA ranged from 92.3% to 100%, with NPA exceeding 99%, and diagnostic accuracy above 99.7%. Viral targets showed PPA from 90.3% to 100% and NPA from 95.8% to 100%. Adenovirus, Influenza A, and Influenza B achieved 100% PPA. RSV and SARS-CoV-2 demonstrated diagnostic accuracies of 99.7% and 99.0%, respectively. Agreement with SOC assays was high (PPA up to 97.1%; kappa 0.81-0.97). Discordance analyses identified differences in pathogen detection, with the Respiratory *Flex* Assay detecting analytes missed by SOC assays. The LIAISON PLEX Respiratory *Flex* Assay demonstrated high accuracy, reliability, and strong diagnostic agreement with SOC assays. Its robust performance across respiratory pathogens supports its use as a comprehensive diagnostic tool for respiratory infection management.

**IMPORTANCE:**

Fast and accurate detection of respiratory pathogens helps guide treatment, lower healthcare costs, and support disease monitoring. The LIAISON PLEX Respiratory *Flex* Assay was evaluated for respiratory pathogen detection by comparing its performance to FDA-cleared molecular respiratory pathogen detection methods, PCR followed by bi-directional sequencing (PCR/BDS) for bacterial pathogens, and standard-of-care (SOC) molecular respiratory assays, including targeted and multiplex syndromic PCR panels. The study assessed its ability to identify pathogens associated with respiratory tract infections, measure agreement with SOC assays, and determine its accuracy in clinical settings. The findings provide data on the assay’s diagnostic performance, testing flexibility, and applicability in laboratory workflows.

## INTRODUCTION

Respiratory tract infections (RTIs) are a major global cause of morbidity and mortality, placing a significant burden on healthcare systems (1, 2). A key diagnostic challenge is the nonspecific and overlapping nature of RTI symptoms. Viral and bacterial pathogens share common indicators like cough, fever, and dyspnea, making it difficult to identify the causative agent based on clinical presentation alone. This diagnostic uncertainty often leads to inappropriate testing and overuse of antibiotics, contributing to the global crisis of antimicrobial resistance (AMR) (3, 4). Overprescription of antibiotics, which are ineffective for viral infections, drives the selection of resistant strains, increases healthcare costs, prolongs hospital stays, and worsens patient outcomes (5–8).

In recent years, syndromic molecular diagnostic panels have transformed RTI management by enabling the simultaneous detection of multiple respiratory pathogens in a single test. These panels improve diagnostic accuracy, reduce turnaround time, and streamline laboratory workflows to support timely clinical decision-making (9, 10). Multiplex testing is particularly valuable during seasonal surges, pandemics, and in complex clinical cases where co-infections are suspected.

In April 2024, Diasorin received FDA clearance for the LIAISON PLEX platform and the Respiratory *Flex* Assay (RSP *Flex*). The system is a fully automated, sample-to-customizable answer system, marking the next generation of the VERIGENE system and the Respiratory Pathogens *Flex* Test (RP *Flex*). The RSP *Flex* assay enables simultaneous qualitative detection of nucleic acids from 14 viral and 5 bacterial respiratory pathogens in nasopharyngeal swabs (NPS), using RT-PCR and nanoparticle-based microarray detection technology. Its customizable design allows laboratories to select clinically relevant targets, supporting workflow flexibility and diagnostic stewardship.

This multicenter study evaluated the clinical performance of the RSP *Flex* assay using NPS specimens from symptomatic patients collected between October 2022 and April 2023 across six geographically diverse clinical sites in the United States as part of an FDA 510(k) submission. Performance was compared to FDA-cleared molecular respiratory assays and PCR followed by bi-directional sequencing (PCR/BDS) for specific bacterial pathogens and benchmarked against standard-of-care (SOC) panels, which included targeted and multiplex syndromic PCR panels commonly available in laboratories.

## MATERIALS AND METHODS

### Clinical specimens

A total of 2,099 samples were collected, including 1,843 clinical specimens in the prospective study analysis and 256 specimens in the retrospective study. The specimens used in this study were remnant samples collected between October 2022 and April 2023. Depending on the time frame for testing, the specimens were either stored refrigerated at 2–8°C if tested within 72 h or frozen at ≤ −70°C if testing was delayed beyond 72 h post-collection. The enrollment period at each site extended from when the first specimen was collected to when the last specimen was collected. All clinical specimens included in this study were collected under IRB-approved protocols at each participating site as part of a U.S. FDA 510(k) submission. A waiver of consent was obtained from each site in accordance with the requirements of each institution’s IRB and the study’s observational design. Demographic and clinical data, including age, gender, geographic location, care setting, and other pertinent clinical details (e.g., symptoms), were collected when available through electronic health record (EHR) abstraction and site-level documentation.

All enrolled patients presented with at least one respiratory symptom at the time of sample collection. The classification of symptom severity into “mild,” “moderate,” and “severe” was based on a combination of clinical presentation and physician-reported assessments. These severity categories were standardized across participating sites using EHR abstraction and clinical documentation to ensure consistency. Mild cases were generally limited to upper respiratory symptoms, such as cough, nasal congestion, and sore throat, without systemic involvement or signs of lower respiratory tract infection. Moderate cases were characterized by systemic features, including fever, chills, or malaise, often accompanied by respiratory complaints, reflecting a more clinically significant illness. Severe cases involved signs of lower respiratory tract involvement, such as shortness of breath or difficulty breathing, frequently accompanied by high fever or multiple systemic symptoms, consistent with more advanced or complicated infection. Severity assignments were intended to reflect both symptom complexity and clinical impact, rather than relying solely on subjective provider labeling at the point of care.

### Prospective specimens

Specimens were collected prospectively from pediatric and adult patients showing clinical signs and symptoms consistent with RTI as determined by a clinician. Specimens were obtained from six geographically diverse clinical sites across the United States, specifically in California (one site), Florida (two sites), Texas (one site), and Wisconsin (two sites). A total of 1,911 unique prospective specimens that met the pre-determined inclusion criteria were enrolled in the study. Of the 1,911 specimens enrolled in the prospective study, 68 were disqualified and removed from further analysis. Most specimen exclusions resulted from non-compliance with the study protocol or failure to meet the inclusion criteria after enrollment. This left a total of 1,843 clinical specimens for evaluation. Among these specimens, 66.3% (*n* = 1,221/1,843) were tested fresh, while 33.7% (*n* = 622/1,843) were tested frozen.

### Retrospective specimens

For targets that exhibited low prevalence rates in the prospective study, the prospective specimen set was supplemented with 256 retrospective remnants (archived and frozen), de-identified specimens sourced from four sites/vendors in the United States. The retrospective specimens were identified by Standard of Care (SOC) and tested using a comparator method following enrollment in the study. To minimize bias, retrospective specimens were randomized and blinded and tested alongside negative specimens at three sites. Retrospective specimen collection dates ranged from May 2016 through June 2023 while testing occurred during August 2023 and December 2023.

### Diasorin LIAISON PLEX Respiratory *Flex* Assay

The LIAISON PLEX RSP *Flex* assay (Diasorin, Austin, TX) is a customizable, syndromic molecular qualitative assay designed for the simultaneous *in vitro* detection and identification of 19 respiratory pathogens, including 14 viral and 5 bacterial targets (Table 1), from NPS collected in Universal Transport Media (UTM) or Universal Viral Transport Media (UVT). This assay is conducted on the automated, sample-to-answer LIAISON PLEX System (Diasorin, Austin, TX), and the assay employs reverse transcription (RT), polymerase chain reaction (PCR), and array hybridization technologies to detect specific nucleic acid sequences from the targeted pathogens within a single, room-temperature disposable test cartridge. Briefly, 300 µL of the NPS sample is added into the sample port of the assay cartridge. The system is initialized by scanning the sample and cartridge IDs, after which the cartridge is loaded onto the LIAISON PLEX System to begin the run. Once the run is completed, the software automatically calculates and displays the results. A positive result indicates the presence of nucleic acid gene sequences for the target in the patient sample, while a negative result signifies that the nucleic acid sequences are either absent or below the assay’s limit of detection. Each cartridge includes internal controls (extraction, amplification, and hybridization controls) to ensure the reliability of sample preparation, amplification, and detection processes.

**TABLE 1:** Targets and reference method algorithm

LIAISON PLEX Respiratory *Flex* Target	Comparator methods
Adenovirus (AdV)	NxTAG Respiratory Pathogen Panel (RPP)
*Chlamydia pneumoniae* (*Cpn*)	
Human coronavirus (hCoV)	
Human enterovirus/rhinovirus (HEV/HRV)	
Human metapneumovirus (hMPV)	
Influenza A (Flu A)	
Influenza A Subtype H1 (Flu A-H1)	
Influenza A Subtype H3 (Flu A-H3)	
Influenza B (Flu B)	
*Mycoplasma pneumoniae* (*Mpn*)	
Human Parainfluenza 1 (HPIV-1)	
Human Parainfluenza 2 (HPIV-2)	
Human Parainfluenza 3 (HPIV-3)	
Human Parainfluenza 4 (HPIV-4)	
Respiratory syncytial virus (RSV)	
*Bordetella holmesii* (*Bh*)	Composite of PCR followed by BDS Nucleic acid amplification test
*Bordetella parapertussis* (*Bpp*)	
*Bordetella pertussis* (*Bp*)	
Severe acute respiratory syndrome coronavirus (SARS-CoV-2)	Simplexa COVID-19 Direct

### Clinical performance against the comparator assays

The LIAISON PLEX RSP *Flex* assay was evaluated for clinical performance by comparing it to the Simplexa COVID-19 Direct assay (Diasorin, Cypress, California) for the SARS-CoV-2 target and the NxTAG Respiratory Pathogen Panel (RPP) assay (Luminex Corporation, Austin, TX) for all other targets, excluding *Bordetella holmesii*, *Bordetella pertussis*, and *Bordetella parapertussis*. Both comparator assays were performed according to their respective manufacturers’ instructions. The selection of these assays was based on FDA requirements for 510(k) submissions, which mandate comparison to existing FDA-cleared assays to establish clinical performance. The Simplexa COVID-19 Direct assay, FDA cleared on 13 September 2022 (K212147), was chosen to satisfy the requirement for an FDA-cleared comparator for the SARS-CoV-2 target. The NxTAG RPP was selected as a broad multiplex panel with overlapping targets and established clinical utility. Both assays are considered standard-of-care platforms and were readily available across participating study sites, ensuring consistent comparator testing and standardized performance evaluation throughout the multicenter study.

Due to the absence of a single FDA-cleared assay covering all three *Bordetella* targets (*B. pertussis*, *B. parapertussis*, and *B. holmesii*), as well as gaps in target coverage and inconsistent implementation across study sites, PCR followed by bi-directional sequencing (BDS) was selected as the comparator method. This composite nucleic acid amplification test (NAAT) method was developed, analytically validated, and qualified for use as a comparator method in support of an FDA 510(k) submission. This reference method was used to evaluate clinical performance of the LIAISON PLEX Respiratory *Flex* Assay for the following targets: *Bordetella holmesii*, *Bordetella parapertussis*, and *Bordetella pertussis*. The composite method employed a two-stage testing algorithm combining multiplexed fragment analysis (FA) with confirmatory bi-directional sequencing (BDS).

Briefly, nucleic acids were extracted from clinical specimens and tested using four multiplexed FA panels. Each panel targeted between one and four analytes using analyte-specific primers labeled at the 5′ end with either HEX or FAM dyes. These primers were designed to generate amplicons with unique fragment sizes, enabling simultaneous detection and differentiation via capillary electrophoresis (CE) on the Applied Biosystems 3730xl Genetic Analyzer. For each analyte, at least two independent primer sets were used to increase detection reliability. If a fragment matching the target-specific size and fluorescence intensity was observed, the specimen was considered FA-positive and advanced to the second stage of testing. FA-positive specimens were subjected to confirmatory BDS. Amplicons were generated using two to three sets of analyte-specific primers, each tagged with universal M13 sequences to facilitate sequencing. Sequence quality was assessed using PHRED scores, with sequences required to contain at least 100 contiguous bases and ≥90% of bases scoring ≥20 to qualify as “good quality.” Good-quality sequences were compared to the NCBI nucleotide database using BLAST. A sequence was considered a positive match to the target if it had a query coverage ≥95%, sequence identity ≥95%, and *e*-value ≤10⁻³⁰. If both forward and reverse strands met these criteria, the specimen was classified as positive for the analyte. If not, or if sequencing quality was insufficient, the result was classified as negative.

Table 1 summarizes the reference method algorithm for each target. Out of the 1,843 clinical specimens included in the evaluation cohort, 1,832 (99.4%) yielded valid results from the Respiratory *Flex* Assay (i.e., “detected” or “not detected”) during the clinical performance studies, resulting in a final invalid rate of 0.6% (*n* = 11/1,843).

### Performance comparison against the standard of care assays

Initial testing using standard of care (SOC) assays was conducted at each study site. The results from these tests were used to enroll positive and negative respiratory specimens into the clinical study. The SOC assays were not included as comparator methods in the performance comparisons for FDA 510(k) clearance of the RSP *Flex* assay. The following FDA-cleared SOC assays: Cepheid Xpert Xpress CoV-2/Flu/RSV Plus (Cepheid; Sunnyvale, California, USA), Abbott Alinity m Resp-4-Plex (Abbott Molecular Inc.; Des Plaines, Illinois, USA), and BioFire Respiratory Panel 2.1 (BioMérieux; Salt Lake City, Utah, USA). All assays were performed according to the manufacturers’ instructions.

### Discordance analysis

For clinical performance for the 510(k) submission, the RSP *Flex* assay result was classified as either true positive (TP) or true negative (TN) only if it matched the results from the comparator methods shown in Table 1. When discrepancies occurred, such as false positive (FP) or false negative (FN) results, an analysis of the discordant results was conducted. These results are not included in the performance calculations but are discussed in the footnotes of the performance tables.

For the SOC comparisons, a discordance analysis was performed using a defined composite reference method (CRM). The CRM included two FDA-cleared assays: the Simplexa COVID-19 Direct assay (Diasorin, Cypress, California), the NxTAG Respiratory Pathogen Panel (RPP) (Luminex Corporation, Austin, TX), and one validated real-time PCR laboratory-developed test (LDT). The LDT was developed, validated internally, and conducted at Diasorin in Austin, TX. The assay targets conserved regions of the *Bordetella* genus and includes species-specific primers and probes for *B. pertussis*, *B. parapertussis*, and *B. holmesii*. The targets were selected based on published sequence data and *in silico* analysis to ensure broad coverage and high specificity while avoiding cross-reactivity. Analytical sensitivity and specificity were confirmed using characterized control materials and previously sequenced clinical isolates. Positive PCR amplification products were further confirmed using bi-directional sequencing (PCR/BDS) of the amplicons to ensure accurate species identification and to resolve discordant findings. Samples were categorized as positive if one or more PCR assays detected respiratory targets, with confirmation from the bi-directional sequencing. Samples were classified as negative if all PCR assays returned negative results. The discordant samples that were not further evaluated by the CRM were excluded due to pre-analytical limitations, primarily insufficient residual sample volume or degraded nucleic acid upon re-extraction. These limitations prevented downstream PCR and sequencing analysis.

### Calculations and statistical analysis

The analysis of Positive Percent Agreement (PPA), Negative Percent Agreement (NPA), and two-sided (upper/lower) 95% confidence interval (CI) was performed using Microsoft Office Excel 365 MSO software (Microsoft, Redmond, WA) and Prism GraphPad software (GraphPad Software Inc., San Diego, CA, USA). The PPA was calculated as TP/(TP + FN) × 100, and the NPA was calculated as TN/(TN + FP) × 100, where TP was true-positive, FN was false-negative, TN was true-negative, and FP was false-positive results. Diagnostic accuracy was calculated as ((TP + TN)/(TN + FP + FN + TP)). Cohen’s kappa coefficient (*κ*) was used to quantify the level of agreement between the LIAISON PLEX Respiratory *Flex* Assay and the selected comparator assays, correcting for agreement expected by chance. The coefficient of the overall agreement was categorized as almost perfect (>0.90), strong (0.80 to 0.90), moderate (0.60 to 0.79), weak (0.40 to 0.59), minimal (0.21 to 0.39), or none (0 to 0.20). The discordance rate was calculated as (FP + FN)/Total number of samples tested × 100.

Study data were generated as part of a clinical study supporting FDA 510(k) clearance (K233410). Relevant summary data are available in the FDA 510(k) database [https://www.fda.gov/]. Additional de-identified data are available upon reasonable request.

## RESULTS

### Demographics and study cohort

A total of 2,099 NPS specimens were analyzed, including 1,843 prospective and 256 retrospective samples from six U.S. sites. The majority of patients were ≤21 years (59%), followed by adults (22–64 years, 27.6%) and the elderly (>65 years, 12.8%) (Table 2). Samples were primarily collected from emergency rooms (43.5%), outpatient settings (28.1%), and hospitalized patients (15.1%).

**TABLE 2:** Demographic overview of study participants (*N* = 2,099)

Demographics	Overall no. (%)
Gender	
Male	956 (45.6%)
Female	1,128 (53.7%)
Unknown	15 (0.7%)
Total	2,099 (100.0%)
Age (years)	
0–1	394 (18.8%)
>1–5	327 (15.6%)
>5–21	516 (24.6%)
22–64	579 (27.6%)
>65	269 (12.8%)
Unknown	14 (0.7%)
Total	2,099 (100.0%)
Subject status	
Emergency room	913 (43.5%)
Hospitalized	317 (15.1%)
Outpatient	590 (28.1%)
Unknown	279 (13.3%)
Total	2,099 (100.0%)

### Symptom distribution and severity

All 1,843 prospective participants presented with at least one respiratory symptom (Fig. S1A). The number of symptoms varied: 603 patients reported three, 514 reported four, 135 reported five, 4 reported six, and 3 reported seven symptoms. Most participants (86.2%; 1,588/1,843) experienced symptom onset within a single day. Fewer patients had symptom progression over 2 days (5.4%; 100/1,843) or 3–7 days (5.8%; 107/1,843). Symptom onset data were unavailable for 2.6% (48/1,843) (Fig. S1B).

Cough was the most frequently reported symptom (71.5%; 1,317/1,843), followed by fever or chills (59.1%; 1,090/1,843) and congestion or runny nose (57.0%; 1,051/1,843). Shortness of breath was reported by 30.0% (553/1,843), sore throat by 28.8% (531/1,843), and body aches by 10.9% (200/1,843). Loss of smell was least common (0.8%; 14/1,843). Additional symptoms (e.g., headache, fatigue, nausea, vomiting) were reported by 33.7% (622/1,843) (Table 3).

**TABLE 3:** Symptom distribution by severity in 1,843 prospective specimens

	Fever or chills	Congestion or runny nose	Cough	Shortness of breath or difficulty breathing	Loss of smell	Sore throat	Body aches	Other
**Severity**								
Mild	199	165	217	86	2	82	16	148
Moderate	28	5	17	14	0	12	0	10
Severe	10	2	7	7	1	6	2	9
Unknown	853	879	1,076	446	11	431	182	455
	**1,090**	**1,051**	**1,317**	**553**	**14**	**531**	**200**	**622**

In mild cases (*n* = 915), cough (23.7%), fever or chills (21.7%), and congestion (18%) were most frequent. Moderate (*n* = 86) and severe cases (*n* = 22) had higher reports of fever and shortness of breath. In cases of unknown severity (*n* = 1,107), cough (97.2%), congestion (79.4%), and fever (77.1%) were most frequently reported (Table 3).

### Pathogen detection and co-detections

Among 1,843 prospective specimens, 1,520 (82.5%) tested positive using the RSP *Flex* Assay (Table 4). HEV/HRV was the most frequently detected pathogen (22.6%), followed by SARS-CoV-2 (16.7%), adenovirus (11.1%), and influenza A (10.0%) (Table 4). Other targets included hMPV (8.6%), HCoV (8.2%), RSV (7.8%), HPIVs 1–4 (4.9%), and influenza B (0.5%). Influenza A subtyping identified Flu A-H1 in 2.5% and Flu A-H3 in 7.1%. Among HPIVs, only HPIV-3 exceeded 1% positivity (2.8%). The only bacterial target detected was *B. parapertussis* (0.5%).

**TABLE 4:** LIAISON PLEX Respiratory *Flex* Assay positive detection rate and co-detection distribution (*n* = 1,520)

	Overall	
Targets	Pos	(%)
Adenovirus (AdV)	168	11.1% (168/1,520)
*Bordetella holmesii* (*Bh*)	0	0.0% (0/1,520)
*Bordetella parapertussis* (*Bpp*)	7	0.5% (7/1,520)
*Bordetella pertussis* (*Bp*)	0	0.0% (0/1,520)
*Chlamydia pneumoniae* (*Cpn*)	0	0.0% (0/1,520)
Human coronavirus (HCoV)	125	8.2% (125/1,520)
Human enterovirus/rhinovirus (HEV/HRV)	344	22.6% (344/1,520)
Human metapneumovirus (hMPV)	131	8.6% (131/1,520)
Influenza A (Flu A)	145	10% (145/1,520)
Influenza A Subtype H1 (Flu A-H1)	38	2.5% (38/1,520)
Influenza A Subtype H3 (Flu A-H3)	108	7.1% (108/1,520)
Influenza B (Flu B)	8	0.5% (8/1,520)
*Mycoplasma pneumoniae* (*Mpn*)	0	0.0% (0/1,520)
Human Parainfluenza 1 (HPIV-1)	11	0.7% (11/1,520)
Human Parainfluenza 2 (HPIV-2)	12	0.8% (12/1,520)
Human Parainfluenza 3 (HPIV-3)	42	2.8% (42/1,520)
Human Parainfluenza 4 (HPIV-4)	9	0.6% (9/1,520)
Respiratory syncytial virus (RSV)	118	7.8% (118/1,520)
Severe acute respiratory syndrome coronavirus (SARS-CoV-2)	254	16.7% (254/1,520)
Total	1,520	100% (1,520/1,520)

Co-detection distribution (*n* = 173/1,520)		
Age distribution		
Pediatric	161	93.1% (161/173)
Adult	10	5.7% (10/173)
Elderly	2	1.2% (2/173)
Pathogen composition		
Viral-viral	156	90.2% (156/173)
Bacterial-viral	17	9.8% (17/173)
Type of co-detection		
Dual	151	87.3% (151/173)
Poly (≥3 pathogens)	22	12.7% (22/173)

Co-detections occurred in 9.4% (173/1,843) of specimens accounting for 11.4% (173/1,520) of positives (Table 4). Most (90.2%; 156/173) were viral-viral combinations; 9.8% (17/173) involved bacterial-viral pairs (Table 4). Co-detections were predominantly seen in pediatric patients (<21 years, 93.1%; 161/173) (Table 4). Dual co-detections represented 87.3% (151/173) of cases, while 12.7% (22/173) involved three or more pathogens (Table 4).

**Fig 1:**
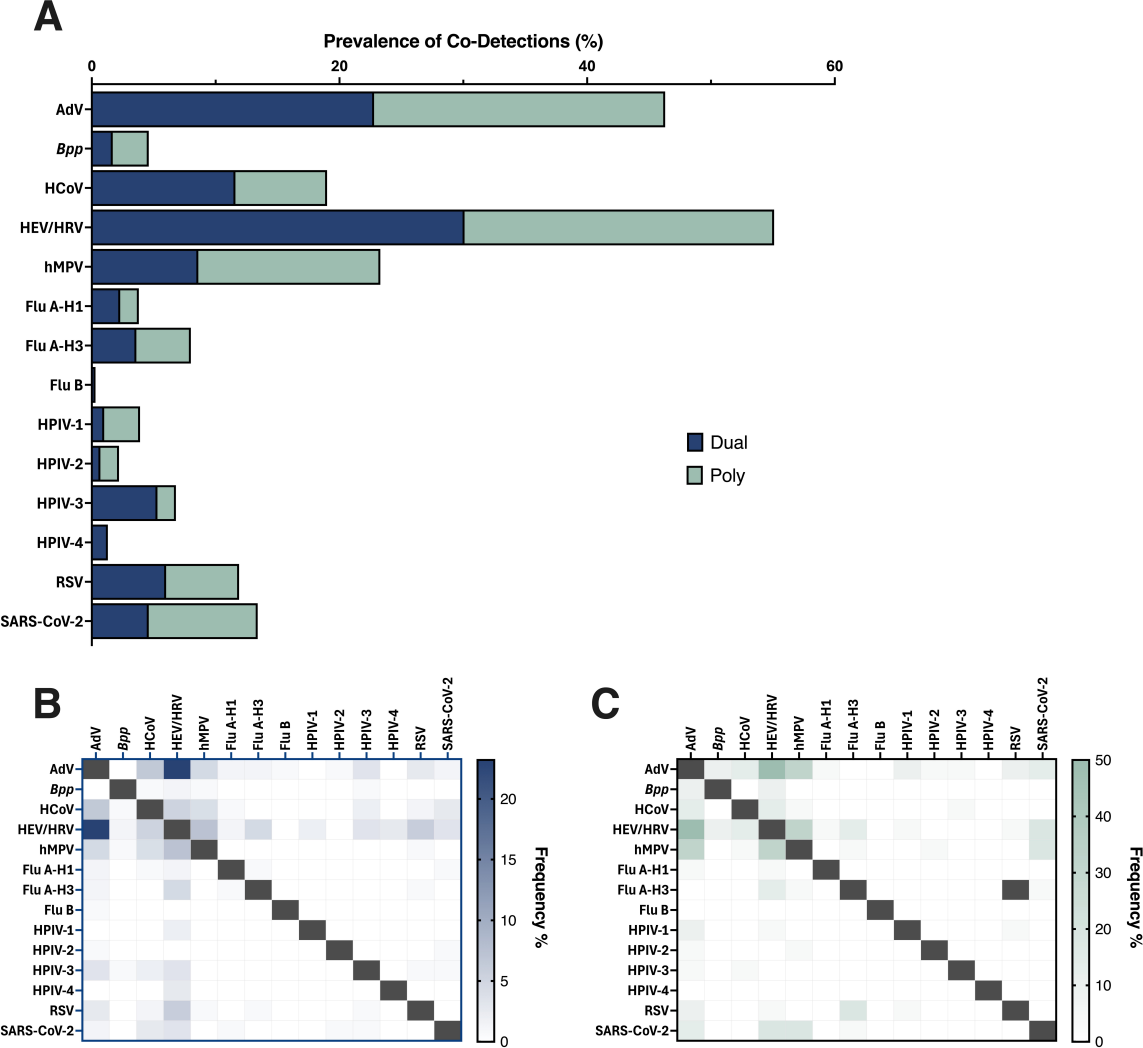
Co-detection prevalence in NP swabs via LIAISON PLEX Respiratory *Flex* Assay. (**A**) Prevalence of specific respiratory targets co-detections categorized as dual (blue) or poly (green) infections; (**B**) Heatmap showing the frequency (%) of dual co-detections among different respiratory pathogens, with darker blue boxes indicating higher co-detection rates; (**C**) Heatmap displaying the frequency (%) of multiple co-detections among various respiratory pathogens, where darker green boxes represent higher co-detection rates between respective pathogen pairs.

HEV/HRV was the most common co-detected pathogen, frequently paired with adenovirus, hMPV, or HCoV (Fig. 1A through C). In dual combinations, HEV/HRV–AdV (23.2%) and HEV/HRV–hMPV (7.3%) were most frequent. In poly co-detections, HEV/HRV was paired with AdV (50.0%) and HCoV (31.8%). *B. parapertussis* was occasionally detected with AdV or HEV/HRV.

### LIAISON PLEX Respiratory *Flex* Assay clinical and comparative performance

The clinical performance of the RSP *Flex* assay was evaluated across 19 respiratory targets, using positive percent agreement (PPA), negative percent agreement (NPA), and diagnostic accuracy (Table 5). For bacterial targets, PPA ranged from 92.3% to 100%, and NPA exceeded 99%. Diagnostic accuracy surpassed 99.7% across most bacterial analytes.

**TABLE 5:** Clinical evaluation of the LIAISON PLEX Respiratory *Flex* Assay vs comparator assays using both prospective and retrospective nasopharyngeal specimens

	Positive percent agreement		Negative percent agreement			Diagnostic accuracy^*a*^
Analytes	TP/(TP + FN)^*b*^	% (±95% CI)^*c*^	TN/(TN + FP)	% (±95% CI)	Total	% (±95% CI)
Bacteria						
*Bordetella holmesii*	0/0	N/A^*d*^	1,964/1,964	100.0% (99.8–100.0)	1,964/1,964	N/A
*Bordetella parapertussis*	12/13^*e*^	92.3% (66.7–98.6)	1,998/2,004^*f*^	99.7% (99.3–99.9)	2,010/2,017	99.7% (99.3–99.9)
*Bordetella pertussis*	23/23	100.0% (85.7–100.0)	1,967/1,970^*g*^	99.8% (99.6–99.9)	1,990/1,993	99.8% (99.6–100.0)
*Chlamydia pneumoniae*	13/14^*e*^	92.9% (68.5–98.7)	2,061/2,062^*g*^	100.0% (99.7–100.0)	2,074/2,076	99.9% (99.7–100.0)
*Mycoplasma pneumoniae*	23/24^*e*^	95.8% (79.8–99.3)	2,046/2,052^*g*^	99.7% (99.4–99.9)	2,069/2,076	99.7% (99.3–99.9)
Viruses						
Adenovirus	100/100	100.0% (96.3–100.0)	1,893/1,976^*h*^	95.8% (94.8–96.6)	1,993/2,076	96.0% (95.1–96.8)
Human coronavirus	121/134^*i*^	90.3% (84.1–94.2)	1,931/1,942^*j*^	99.4% (99.0–99.7)	2,052/2,076	98.8% (98.3–99.3)
Human enterovirus/rhinovirus	335/359^*k*^	93.3% (90.2–95.5)	1,678/1717^*l*^	97.7% (96.9–98.3)	2,013/2,076	97.0% (96.1–97.7)
Human metapneumovirus	126/132^*m*^	95.5% (90.4–97.9)	1,938/1,944^*n*^	99.7% (99.3–99.9)	2,064/2,076	99.4% (99.0–99.7)
Influenza A	130/130	100.0% (97.1–100.0)	1,929/1,946^*o*^	99.1% (98.6–99.5)	2,059/2,076	99.2% (98.7–99.5)
Influenza A Subtype H1	38/38	100.0% (90.8–100.0)	2,036/2,038^*p*^	99.9% (99.6–100.0)	2,074/2,076	99.9% (99.7–100.0)
Influenza A Subtype H3	104/107^*q*^	97.2% (92.1–99.0)	1,965/1,969^*r*^	99.8% (99.5–99.9)	2,069/2,076	99.7% (99.3–99.9)
Influenza B	31/31	100.0% (89.0–100.0)	2,044/2,045^*g*^	100.0% (99.7–100.0)	2,075/2,076	99.9% (99.7–100.0)
Human Parainfluenza 1	29/30^*e*^	96.7% (83.3–99.4)	2,045/2,046^*g*^	100.0% (99.7–100.0)	2,074/2,076	99.9% (99.7–100.0)
Human Parainfluenza 2	31/33^*e*^^,^^*g*^	93.9% (80.4–98.3)	2,042/2,043^*g*^	100.0% (99.7–100.0)	2,073/2,076	99.9% (99.6–100.0)
Human Parainfluenza 3	43/46^*s*^	93.5% (82.5–97.8)	2,029/2,030^*t*^	100.0% (99.7–100.0)	2,072/2,076	99.8% (99.5–100.0)
Human Parainfluenza 4	31/32^*e*^	96.9% (84.3–99.4)	2,040/2,044^*u*^	99.8% (99.5–99.9)	2,071/2,076	99.8% (99.4–99.9)
Respiratory syncytial virus	127/132^*v*^	96.2% (91.4–98.4)	1,943/1,944^*g*^	99.9% (99.7–100.0)	2,070/2,076	99.7% (99.4–99.9)
SARS-CoV-2	246/255^*w*^	96.5% (93.4–98.1)	1,517/1,525^*x*^	99.5% (99.0–99.7)	1,763/1,780	99.0% (98.5–99.4)

^*a*^ Diagnostic Accuracy = ((TP + TN) / (TN + FP + FN + TP)).

^*b*^ TP, true positive; FN, false negative; TN, true negative; FP, false positive.

^*c*^ ±, upper/lower 95% CI, confidence interval.

^*d*^ Not applicable.

^*e*^ One False Negative specimen by the Respiratory *Flex* Assay was positive by PCR/BDS.

^*f*^ Of the 6 specimens with false positive *Bordetella parapertussis *results by the Respiratory *Flex* Aassay, 1 was negative by an FDA-cleared molecular respiratory panel and 5 were not tested.

^*g*^ The comparator analysis resulted in a specimen without valid reference results for this target.

^*h*^ Of the 83 specimens with false positive adenovirus results by the Respiratory Flex Aassay, 21 were positive by an FDA-cleared molecular respiratory panel, 28 were negative, and 34 were not tested.

^*i*^ Of the 13 specimens with false negative coronavirus results by the Respiratory Flex Aassay, 3 were negative by PCR/BDS, 9 were positive, and 1 was not tested.

^*j*^ Of the 11 specimens with false positive coronavirus results by the Respiratory Flex Aassay, 5 were positive by PCR/BDS, 2 were negative, 3 did not produce results, and 1 was not tested.

^*k*^ Of the 24 specimens with false negative enterovirus/rhinovirus results by the Respiratory Flex Aassay, 10 were positive by PCR/BDS, 9 were negative, and 5 were not tested.

^*l*^ Of the 39 specimens with false positive enterovirus/rhinovirus results by the Respiratory Flex Assay, 4 were positive by PCR/BDS, 31 were negative, and 4 were not tested.

^*m*^ Of the 6 specimens with false negative hPMV results by the Respiratory Flex Aassay, 4 were positive by PCR/BDS and 2 were negative.

^*n*^ Of the 6 specimens with false positive hPMV results by the Respiratory Flex Assay, 4 were positive by PCR/BDS and 2 were negative.

^*o*^ Of the 17 specimens with false positive influenza A results by the Respiratory Flex Assay, 7 were positive by PCR/BDS and 10 were negative.

^*p*^ One Influenza A H1 False Positive was negative by PCR/BDS and one was not tested.

^*q*^ All three Influenza A H3 False Negatives were negative by PCR/BDS.

^*r*^ All four Influenza A H3 False Positives were negative by PCR/BDS.

^*s*^ Of the 3 specimens with false negative parainfluenza 3 results by the Respiratory Flex Assay, 2 were negative by PCR/BDS and 1 was not tested.

^*t*^ One False Positive specimen by the Respiratory *Flex* Assay was negative by PCR/BDS.

^*u*^ All four Parainfluenza 4 False Positives were negative by PCR/BDS.

^*v*^ Of the 5 specimens with false negative RSV results by the Respiratory Flex Assay, 1 was negative by PCR/BDS, and 3 were negative by an FDA-cleared molecular Flu/RSV assay.

^*w*^ Of the 9 specimens with false negative SARS-CoV-2 results by the Respiratory Flex Assay, 5 were positive by PCR/BDS, 2 were negative, and 2 were not tested.

^*x*^ Of the 8 specimens with false positive SARS-CoV-2 results by the Respiratory Flex Assay, 5 were positive by PCR/BDS, 2 were negative, and 1 was not tested.

For viral targets, PPA ranged from 90.3% to 100%, and NPA from 95.8% to 100%. Adenovirus, Influenza A, Influenza B, and Parainfluenza 1 showed 100% PPA. RSV and SARS-CoV-2 achieved diagnostic accuracy of 99.7% and 99.0%, respectively.

Comparative performance was assessed against three FDA-cleared syndromic panels: Cepheid Xpert Xpress CoV-2/Flu/RSV Plus, Abbott Alinity m Resp-4-Plex, and BioFire RP 2.1 (Table 6). Key metrics included PPA, NPA, kappa coefficient (*κ*), and discordance rates.

**TABLE 6:** Performance evaluation of the LIAISON PLEX Respiratory *Flex* Assay vs SOC assays using nasopharyngeal specimens

	LIAISON PLEX RP Flex		(± 95% CI)^*a*^^,*b*^			
SOC assay^*c*^	Positive	Negative	PPA^*d*^	NPA^*e*^	Kappa (*κ*)^*f*^	Discordance rate (%)
Targeted assays						
Cepheid Xpert CoV-2/Flu/RSV						
Positive	67	1^*g*^	97.1%	99.2%	0.97	1.6%
Negative	2^*h*^	122	(0.90–0.99)	(0.96–1.0)	(0.93–1.0)	
Abbott Alinity m						
Positive	22	1^*i*^	95.7%	97.4%	0.9	3.2%
Negative	1^*j*^	38	(0.78–0.99)	(0.86–0.99)	(0.79–1.0)	
Syndromic assay						
BioFire RP 2.1						
Positive	141	12^*k*^	92.8%	88.2%	0.81	9.1%
Negative	11^*l*^	90	(0.87–0.96)	(0.80–0.94)	(0.74–0.89)	

^*a*^ ±, upper/lower 95%.

^*b*^ CI, confidence interval.

^*c*^ Standad of care assays.

^*d*^ Positive percent agreement (PPA).

^*e*^ Negative percent agreement (NPA).

^*f*^ Almost-perfect (>0.90), strong (0.80 to 0.90), moderate (0.60 to 0.79), weak (0.40 to 0.59), minimal (0.21 to 0.39), or none (0 to 0.20).

^*g*^ One LIAISON PLEX negative sample was identified as SARS-CoV-2 positive by the Cepheid assay.

^*h*^ Both positive samples from LIAISON PLEX were not detected by the Cepheid assay.

^*i*^ One LIAISON PLEX negative sample was identified as SARS-CoV-2 positive by Abbott assay.

^*j*^ One Influenza B positive LIAISON PLEX sample was not detected by the Abbott assay.

^*k*^ Out of the 12 LIAISON PLEX negative samples, BioFire identified 6 HEV/HRV, 2 SARS-CoV-2, 2 RSV, one HPIV-3, and 1 influenza A.

^*l*^ Of the 11 LIAISON PLEX positive results, 6 were not detected by BioFire and 5 samples detected different analytes between the 2 assays.

Against Cepheid, RSP *Flex* showed PPA of 97.1% and NPA of 99.2% (*n* = 192), with *κ* = 0.97 and a 1.6% discordance rate. CRM confirmed two Cepheid discordant results: one RSV missed by Cepheid and one SARS-CoV-2 confirmed positive by both. One RSP *Flex* SARS-CoV-2 positive was a false positive.

In the Abbott comparison, RSP *Flex* had a PPA of 95.7%, an NPA of 97.4% (*n* = 62), a *κ* = 0.90, and a 3.2% discordance. CRM confirmed one SARS-CoV-2 positive missed by RSP *Flex* and one Flu B detected by RSP *Flex*.

Compared with BioFire RP 2.1, RSP *Flex* achieved PPA of 92.8%, NPA of 88.2% (*n* = 254), *κ* = 0.81, and 9.1% discordance. Among 23 discordant samples, 12 were positive by BioFire but negative by RSP *Flex*, and 11 were the reverse. CRM classified eight BioFire positives as false positives and confirmed four as true positives. Of the 11 RSP *Flex*-positive samples, five were validated by CRM. One sample was dual positive (RSV and HEV/HRV) by CRM, matching both assay results. Overall, for the BioFire discordant samples, the CRM confirmed eight RSP *Flex* true negatives, six true positives, four false negatives, and five false positives (Table S2).

## DISCUSSION

Fast, accurate detection of respiratory pathogens is essential for guiding treatment, lowering healthcare costs, and supporting disease surveillance. This multicenter study evaluated prospectively collected NPS specimens from a large, geographically diverse cohort in the United States. Our findings offer insight into demographic trends, common symptoms, pathogen detection patterns, and the clinical performance of the newly FDA-cleared, customizable LIAISON PLEX Respiratory *Flex* Assay compared to existing SOC assays. This discussion highlights critical considerations for effective respiratory pathogen identification and underscores the importance of reliable, high-throughput, and customizable assays in modern clinical practice.

### Demographics and symptom presentation

A central observation was the greater prevalence of respiratory infections among individuals aged ≤21 years, who represented 59% of the study population, consistent with reports of increased pathogen exposure in communal settings (11, 12). Their developing immune systems and frequent contact with infected peers likely contribute to this heightened burden of infection.

A slightly higher proportion of participants were female (53.7%), aligning with studies noting greater healthcare utilization among women (13). Hormonal and immunological differences may also influence susceptibility and immune response between genders (14).

Cough, fever/chills, and congestion were the most commonly reported symptoms, reflecting typical viral respiratory illness presentations (15). Mild cases were primarily associated with cough, while moderate-to-severe cases were more frequently associated with shortness of breath and fever/chills, features commonly linked to more advanced respiratory involvement (16, 17). These findings underscore the clinical need for accurate diagnostic tools that support pathogen identification across a range of symptom profiles and patient populations.

### Pathogen detection, co-detections, and clinical implications

Among the pathogens tested, HEV/HRV had the highest detection rate at 22.6%, consistent with its established role as a primary cause of upper RTIs, particularly in pediatric and immunocompromised individuals (18). Although HEV/HRV infections are often regarded as mild, they can exacerbate conditions such as asthma and chronic obstructive pulmonary disease (COPD), underscoring the importance of rapid and precise detection, especially in vulnerable populations (19, 20). Immunocompromised patients are at risk for protracted illness and secondary complications when infected with HEV/HRV, highlighting the need for heightened surveillance and careful clinical management (21). Therefore, interpretation of HEV/HRV detection requires integrating molecular findings with symptomatology, disease severity, immune status, and timing of presentation to ensure appropriate management of patients.

SARS-CoV-2 was detected in 16.7% of symptomatic individuals, illustrating its continued circulation despite vaccination programs and antiviral therapies. Co-circulation of SARS-CoV-2 and other respiratory pathogens poses diagnostic and public health challenges, particularly for differentiating COVID-19 from illnesses caused by other viruses (22). Co-infections involving SARS-CoV-2 and other pathogens have been described, potentially leading to more severe disease or prolonged viral shedding (23, 24).

Bacterial pathogens were detected at lower rates compared to viral targets, which is consistent with sample collection during the peak viral respiratory season (25–27). *B. parapertussis* was identified in 0.5% of samples, aligning with the reemergence observed in similar regions (28, 29). Bacterial infections often follow viral illness, and patients in outpatient settings are typically tested early in their disease course to identify viral pathogens; bacterial infections may not yet have manifested at that time, contributing to lower detection rates (29–31). The ability of the LIAISON PLEX Respiratory *Flex* Assay to detect possible co-infections, even at low bacterial incidence, highlights its comprehensive utility and underscores the value of broad coverage to guide clinical decision-making and antimicrobial stewardship.

Co-detections were detected in 9.4% of patients, mainly dual viral infections. HEV/HRV was frequently co-detected with Adenovirus, human coronaviruses, and RSV. These co-detection profiles may, in part, reflect the high individual prevalence of these respiratory viruses, which increases the likelihood of their co-detection within the same patient. Nonetheless, this pattern is consistent with existing literature reporting high rates of viral co-infection, particularly among pediatric and immunocompromised patients (32, 33).

While the clinical impact of co-infections remains variable, several studies have suggested that certain viral combinations, such as RSV and HRV, may be associated with more severe outcomes, including prolonged hospitalization and greater need for respiratory support (32, 33). In this cohort, no statistical analyses were performed to evaluate the significance of pathogen-pathogen interactions or their association with disease severity.

Nonetheless, the ability to detect co-infections remains clinically important. Accurate and comprehensive molecular diagnostics enable the early recognition of multi-pathogen infections, support more tailored patient management, and help reduce unnecessary antibiotic use by distinguishing between viral and bacterial etiologies. Future studies with larger and more diverse cohorts will be warranted to explore these relationships further using appropriate statistical modeling approaches.

### Clinical performance of the LIAISON PLEX Respiratory *Flex* Assay

Accurate and timely detection of respiratory pathogens is essential for preventing misdiagnoses, informing appropriate treatment, and minimizing complications, especially in individuals with underlying health conditions or compromised immune function. Rapid identification of the infectious etiology supports clinical decision-making, potentially reducing unnecessary antibiotic use and hospital admissions, while contributing to diagnostic and antimicrobial stewardship efforts.

The LIAISON PLEX Respiratory *Flex* Assay demonstrated a diagnostic accuracy exceeding 97% for both viral and bacterial respiratory targets, matching the performance of the SOC assays evaluated in this study. Despite this strong overall performance, modest reductions in specificity for adenovirus and sensitivity for human coronavirus (HCoV) and enterovirus/rhinovirus (HEV/HRV) were observed. For adenovirus, reduced specificity may reflect asymptomatic carriage and prolonged viral shedding, particularly in pediatric and immunocompromised populations, which complicates interpretation (34, 35). Its extensive serotype diversity, over 100 genetically distinct strains, adds further complexity to detection (36). Similarly, the broad genetic heterogeneity of HEV/HRV and endemic HCoVs (229E, NL63, OC43, and HKU1) presents challenges to consistent assay sensitivity (37–39).

Assay design influences these performance variations. Targeting highly conserved regions such as the 5′ untranslated region (UTR) can improve inclusivity but may reduce specificity due to sequence overlap across serotypes (40). Conversely, structural genes offer greater specificity but may miss divergent or emerging strains (40). These trade-offs are inherent to multiplexed syndromic panels, where primer/probe competition and thermodynamic interactions can impact amplification efficiency, particularly for genetically diverse targets (41, 42).

Nevertheless, despite these modest variations in target-specific performance, the assay maintained high overall diagnostic agreement and accuracy across all analytes. Positive and negative percent agreements remained well within clinically acceptable thresholds. The assay’s performance demonstrated strong concordance with SOC assays and was consistent with the diagnostic benchmarks established by other syndromic molecular panels routinely employed in respiratory pathogen detection and surveillance.

### Conclusion

The diverse etiology of respiratory infections observed in this study highlights the critical role of molecular diagnostics in improving detection accuracy and efficiency. Future studies should further clarify how multiple infections influence disease severity and treatment outcomes, particularly in high-risk populations. The prevalence of co-infections and overlapping presentations underscores the need for broad diagnostic coverage. Although not evaluated here, the RSP *Flex* assay’s customizable features warrant exploration to address seasonal variation, risk stratification, and cost considerations. Aligning test selection with diagnostic stewardship principles and epidemiologic data can help optimize laboratory resources and diagnostic yield.

In summary, the LIAISON PLEX Respiratory *Flex* Assay demonstrates high clinical reliability and agreement with SOC assays, supporting its use in respiratory pathogen detection. Its broad pathogen coverage and customizable design align with current diagnostic stewardship priorities, making it a valuable tool for enhancing patient care, reducing healthcare costs, and streamlining diagnostic workflows within a single testing platform.

## SUPPLEMENTAL MATERIAL

Supplemental figures jcm.00708-25-s0001.tiff Figure S1.

Supplemental tables jcm.00708-25-s0002.docx Tables S1 and S2.

ASM does not own the copyrights to Supplemental Material that may be linked to, or accessed through, an article. The authors have granted ASM a non-exclusive, world-wide license to publish the Supplemental Material files. Please contact the corresponding author directly for reuse.
