# Supplementary material for: Multicenter clinical performance and comparative evaluation of the LIAISON PLEX Respiratory Flex Assay for syndromic detection of viral and bacterial pathogens in nasopharyngeal swabs
Source: J Clin Microbiol. 2025 Nov 5;63(12):e00708-25. doi: 10.1128/jcm.00708-25 (PMC12710336; doi:10.1128/jcm.00708-25)
Supplement: Supplemental tables — Tables S1 and S2. [file jcm.00708-25-s0002.docx]

**Table S1.** Co-Detections by the LIAISON PLEX Respiratory *Flex* Assay (*n* = 173).

|  | **Overall Cohort (N=1,520)** | | |
| --- | --- | --- | --- |
| **Co-detections** | **#POS** | **N** | **%** |
| Adenovirus (A / B / C / D / E / F) \| Bordetella parapertussis \| Enterovirus/Rhinovirus | 2 | 173 | 1.16% |
| Adenovirus (A / B / C / D / E / F) \| Coronavirus (HKU1 / 229E / NL63 / OC43) | 10 | 173 | 5.78% |
| Adenovirus (A / B / C / D / E / F) \| Coronavirus (HKU1 / 229E / NL63 / OC43) \| Enterovirus/Rhinovirus | 1 | 173 | 0.58% |
| Adenovirus (A / B / C / D / E / F) \| Coronavirus (HKU1 / 229E / NL63 / OC43) \| Human Metapneumovirus | 1 | 173 | 0.58% |
| Adenovirus (A / B / C / D / E / F) \| Coronavirus (HKU1 / 229E / NL63 / OC43) \| Parainfluenza 3 | 1 | 173 | 0.58% |
| Adenovirus (A / B / C / D / E / F) \| Enterovirus/Rhinovirus | 35 | 173 | 20.23% |
| Adenovirus (A / B / C / D / E / F) \| Enterovirus/Rhinovirus \| Human Metapneumovirus | 3 | 173 | 1.73% |
| Adenovirus (A / B / C / D / E / F) \| Enterovirus/Rhinovirus \| Human Metapneumovirus \| SARS-CoV-2 | 1 | 173 | 0.58% |
| Adenovirus (A / B / C / D / E / F) \| Enterovirus/Rhinovirus \| Influenza A \| Influenza A (subtype H1) | 1 | 173 | 0.58% |
| Adenovirus (A / B / C / D / E / F) \| Enterovirus/Rhinovirus \| Parainfluenza 1 | 1 | 173 | 0.58% |
| Adenovirus (A / B / C / D / E / F) \| Enterovirus/Rhinovirus \| Respiratory Syncytial Virus (RSV A and RSV B) | 1 | 173 | 0.58% |
| Adenovirus (A / B / C / D / E / F) \| Enterovirus/Rhinovirus \| SARS-CoV-2 | 1 | 173 | 0.58% |
| Adenovirus (A / B / C / D / E / F) \| Human Metapneumovirus | 7 | 173 | 4.05% |
| Adenovirus (A / B / C / D / E / F) \| Human Metapneumovirus \| Parainfluenza 2 | 1 | 173 | 0.58% |
| Adenovirus (A / B / C / D / E / F) \| Human Metapneumovirus \| SARS-CoV-2 | 1 | 173 | 0.58% |
| Adenovirus (A / B / C / D / E / F) \| Influenza A \| Influenza A (subtype H1) | 2 | 173 | 1.16% |
| Adenovirus (A / B / C / D / E / F) \| Influenza A \| Influenza A (subtype H3) | 2 | 173 | 1.16% |
| Adenovirus (A / B / C / D / E / F) \| Influenza B | 1 | 173 | 0.58% |
| Adenovirus (A / B / C / D / E / F) \| Parainfluenza 1 \| Respiratory Syncytial Virus (RSV A and RSV B) | 1 | 173 | 0.58% |
| Adenovirus (A / B / C / D / E / F) \| Parainfluenza 2 | 1 | 173 | 0.58% |
| Adenovirus (A / B / C / D / E / F) \| Parainfluenza 3 | 5 | 173 | 2.89% |
| Adenovirus (A / B / C / D / E / F) \| Respiratory Syncytial Virus (RSV A and RSV B) | 4 | 173 | 2.31% |
| Adenovirus (A / B / C / D / E / F) \| SARS-CoV-2 | 2 | 173 | 1.16% |
| Bordetella parapertussis \| Coronavirus (HKU1 / 229E / NL63 / OC43) | 1 | 173 | 0.58% |
| Bordetella parapertussis \| Enterovirus/Rhinovirus | 2 | 173 | 1.16% |
| Bordetella parapertussis \| Human Metapneumovirus | 1 | 173 | 0.58% |
| Bordetella parapertussis \| Parainfluenza 3 | 1 | 173 | 0.58% |
| Coronavirus (HKU1 / 229E / NL63 / OC43) \| Enterovirus/Rhinovirus | 8 | 173 | 4.62% |
| Coronavirus (HKU1 / 229E / NL63 / OC43) \| Enterovirus/Rhinovirus \| Human Metapneumovirus | 1 | 173 | 0.58% |
| Coronavirus (HKU1 / 229E / NL63 / OC43) \| Enterovirus/Rhinovirus \| SARS-CoV-2 | 1 | 173 | 0.58% |
| Coronavirus (HKU1 / 229E / NL63 / OC43) \| Human Metapneumovirus | 6 | 173 | 3.47% |
| Coronavirus (HKU1 / 229E / NL63 / OC43) \| Influenza A \| Influenza A (subtype H1) | 1 | 173 | 0.58% |
| Coronavirus (HKU1 / 229E / NL63 / OC43) \| Parainfluenza 3 | 3 | 173 | 1.73% |
| Coronavirus (HKU1 / 229E / NL63 / OC43) \| Respiratory Syncytial Virus (RSV A and RSV B) | 2 | 173 | 1.16% |
| Coronavirus (HKU1 / 229E / NL63 / OC43) \| SARS-CoV-2 | 4 | 173 | 2.31% |
| Enterovirus/Rhinovirus \| Human Metapneumovirus | 11 | 173 | 6.36% |
| Enterovirus/Rhinovirus \| Human Metapneumovirus \| Influenza A (subtype H3) \| SARS-CoV-2 | 1 | 173 | 0.58% |
| Enterovirus/Rhinovirus \| Human Metapneumovirus \| SARS-CoV-2 | 1 | 173 | 0.58% |
| Enterovirus/Rhinovirus \| Influenza A \| Influenza A (subtype H1) | 2 | 173 | 1.16% |
| Enterovirus/Rhinovirus \| Influenza A \| Influenza A (subtype H3) | 7 | 173 | 4.05% |
| Enterovirus/Rhinovirus \| Influenza A \| Influenza A (subtype H3) \| Respiratory Syncytial Virus (RSV A and RSV B) | 2 | 173 | 1.16% |
| Enterovirus/Rhinovirus \| Parainfluenza 1 | 3 | 173 | 1.73% |
| Enterovirus/Rhinovirus \| Parainfluenza 3 | 5 | 173 | 2.89% |
| Enterovirus/Rhinovirus \| Parainfluenza 4 | 4 | 173 | 2.31% |
| Enterovirus/Rhinovirus \| Respiratory Syncytial Virus (RSV A and RSV B) | 9 | 173 | 5.20% |
| Enterovirus/Rhinovirus \| SARS-CoV-2 | 5 | 173 | 2.89% |
| Human Metapneumovirus \| Respiratory Syncytial Virus (RSV A and RSV B) | 1 | 173 | 0.58% |
| Influenza A \| Influenza A (subtype H1) \| Influenza A (subtype H3) | 1 | 173 | 0.58% |
| Influenza A \| Influenza A (subtype H1) \| SARS-CoV-2 | 1 | 173 | 0.58% |
| Influenza A \| Influenza A (subtype H3) \| Respiratory Syncytial Virus (RSV A and RSV B) | 1 | 173 | 0.58% |
| Parainfluenza 2 \| SARS-CoV-2 | 1 | 173 | 0.58% |
| Parainfluenza 3 \| Respiratory Syncytial Virus (RSV A and RSV B) | 1 | 173 | 0.58% |
| Parainfluenza 3 \| SARS-CoV-2 | 1 | 173 | 0.58% |
| **Total** | 173 |  | 100.00% |

**Table S2.** Analysis of Discordant Samples for the LIAISON PLEX® Respiratory *Flex* Assay Compared to the Standard of Care (SOC) Assays.

| **LIAISON PLEX^®^ RP *Flex*** | **SOC^a^** | **CRM^b^** | **Comments^c^** |
| --- | --- | --- | --- |
|  | **Cepheid** |  |  |
| Not Detected | SARS-CoV-2 | SARS-CoV-2 | FN |
| RSV | Not Detected | RSV | TP |
| SARS-CoV-2 | Not Detected | Not Detected | FP |
|  | **Abbott** |  |  |
| Not Detected | SARS-CoV-2 | SARS-CoV-2 | FN |
| Influenza B | Influenza A | Influenza B | TP |
|  | **BioFire** |  |  |
| Not Detected | SARS-CoV-2 | Not Detected | TN |
| Not Detected | SARS-CoV-2 | Not Detected | TN |
| Not Detected | HEV/HRV | Not Detected | TN |
| Not Detected | HEV/HRV | Not Detected | TN |
| Not Detected | HEV/HRV | Not Detected | TN |
| Not Detected | RSV | Not Detected | TN |
| Not Detected | RSV | Not Detected | TN |
| Not Detected | HPIV-3 | Not Detected | TN |
| Not Detected | HEV/HRV | HEV/HRV | FN |
| Not Detected | HEV/HRV | HEV/HRV | FN |
| Not Detected | HEV/HRV | HEV/HRV | FN |
| Not Detected | Influenza A | Influenza A | FN |
| AdV | Not Detected | Not Detected | FP |
| AdV | Not Detected | Not Detected | FP |
| AdV | HEV/HRV | HEV/HRV | FP |
| HEV/HRV | Not Detected | Not Detected | FP |
| HEV/HRV | Not Detected | Not Detected | FP |
| AdV \| HEV/HRV | Not Detected | AdV \| HEV/HRV | TP |
| Influenza B | Influenza A | Influenza B | TP |
| RSV | HCoV | RSV | TP |
| HEV/HRV | SARS-CoV-2 | HEV/HRV | TP |
| HEV/HRV | Not Detected | HEV/HRV | TP |
| RSV | HEV/HRV | RSV \| HEV/HRV | TP for both |
| ^a^ Standard of care results  ^b^ Composite reference method  ^c^ True positive (TP), true negative (TN), false positive (FP), false negative (FN) for LIAISON PLEX RP *Flex* | | | |

**Fig.S1 Distribution of Number of Symptoms and Symptoms within 7 days Onset in Analyzed Prospective Specimens. A.** The distribution of study participants was based on the number of reported symptoms, ranging from one to seven. The x-axis represents the number of participants, while the y-axis indicates the number of symptoms reported.; **B.** Distribution of study participants based on the number of days after symptom onset, up to 7 days. The x-axis represents the number of participants, and the y-axis shows the symptom onset range (in days). "UNK" represents participants with an unknown symptom onset range.
